# The Salzburg 10/7 HIIT shock cycle study: the effects of a 7-day high-intensity interval training shock microcycle with or without additional low-intensity training on endurance performance, well-being, stress and recovery in endurance trained athletes—study protocol of a randomized controlled trial

**DOI:** 10.1186/s13102-022-00456-8

**Published:** 2022-05-07

**Authors:** Thomas Leonhard Stöggl, Julia C. Blumkaitis, Tilmann Strepp, Mahdi Sareban, Perikles Simon, Elmo W. I. Neuberger, Thomas Finkenzeller, Natalia Nunes, Lorenz Aglas, Nils Haller

**Affiliations:** 1 Department of Sport and Exercise Science, University of Salzburg, Salzburg, Austria; 2 Red Bull Athlete Performance Center, Salzburg, Austria; 3 University Institute of Sports Medicine, Prevention and Rehabilitation and Research Institute of Molecular Sports Medicine and Rehabilitation, Paracelsus Medical University, Salzburg, Austria; 4 Department of Sports Medicine, Rehabilitation and Disease Prevention, Johannes Gutenberg University of Mainz, Mainz, Germany; 5 Department of Biosciences, University of Salzburg, Salzburg, Austria; 6 Genetics Division, Department of Morphology and Genetics, Universidade Federal de São Paulo, São Paulo, Brazil

**Keywords:** Block training, HIT, HIIT, Interval exercise, LIT, Load monitoring

## Abstract

**Background:**

Performing multiple high-intensity interval training (HIIT) sessions in a compressed period of time (approximately 7–14 days) is called a HIIT shock microcycle (SM) and promises a rapid increase in endurance performance. However, the efficacy of HIIT-SM, as well as knowledge about optimal training volumes during a SM in the endurance-trained population have not been adequately investigated. This study aims to examine the effects of two different types of HIIT-SM (with or without additional low-intensity training (LIT)) compared to a control group (CG) on key endurance performance variables. Moreover, participants are closely monitored for stress, fatigue, recovery, and sleep before, during and after the intervention using innovative biomarkers, questionnaires, and wearable devices.

**Methods:**

This is a study protocol of a randomized controlled trial that includes the results of a pilot participant. Thirty-six endurance trained athletes will be recruited and randomly assigned to either a HIIT-SM (HSM) group, HIIT-SM with additional LIT (HSM + LIT) group or a CG. All participants will be monitored before (9 days), during (7 days), and after (14 days) a 7-day intervention, for a total of 30 days. Participants in both intervention groups will complete 10 HIIT sessions over 7 consecutive days, with an additional 30 min of LIT in the HSM + LIT group. HIIT sessions consist of aerobic HIIT, i.e., 5 × 4 min at 90–95% of maximal heart rate interspersed by recovery periods of 2.5 min. To determine the effects of the intervention, physiological exercise testing, and a 5 km time trial will be conducted before and after the intervention.

**Results:**

The feasibility study indicates good adherence and performance improvement of the pilot participant. Load monitoring tools, i.e., biomarkers and questionnaires showed increased values during the intervention period, indicating sensitive variables.

**Conclusion:**

This study will be the first to examine the effects of different total training volumes of HIIT-SM, especially the combination of LIT and HIIT in the HSM + LIT group. In addition, different assessments to monitor the athletes' load during such an exhaustive training period will allow the identification of load monitoring tools such as innovative biomarkers, questionnaires, and wearable technology.

*Trial Registration*: clinicaltrials.gov, NCT05067426. Registered 05 October 2021—Retrospectively registered, https://clinicaltrials.gov/ct2/show/NCT05067426. *Protocol Version* Issue date: 1 Dec 2021. Original protocol. Authors: TLS, NH.

**Supplementary Information:**

The online version contains supplementary material available at 10.1186/s13102-022-00456-8.

## Background

Athletes use high-intensity interval training (HIIT) as a complementary training method to continuous endurance training [1]. HIIT sessions are usually less time-consuming but still effective to improve the aerobic fitness compared with high volume low-intensity sessions [2–6]. HIIT consists of repeated bouts of high-intensity efforts followed by active or passive recovery periods of varying duration (e.g., 4 bouts of 4-min (min) intervals with 3 min rest) which usually results in high levels of metabolic stress, and neuromuscular as well as musculoskeletal load [7, 8]. Therefore, it is recommended to separate HIIT sessions 48 h from each other to ensure adequate recovery [8].

Nevertheless, a "HIIT shock microcycle (HIIT-SM)” approach has been increasingly applied in recent years, i.e., repeated HIIT sessions in a compressed period of time (e.g., 15 HIIT sessions in 11 days) [9]. Studies have examined the effects of isolated (i.e., a “stand-alone” HIIT-SM, not part of further training periodization) HIIT-SM [9–13] as well as HIIT-SM integrated into block periodization [14–16]. Isolated HIIT-SM have been performed between 9 and 21 days, during which 9 to 24 sessions were conducted [9–13, 17–19]. This concept is discussed to promise a rapid increase in performance, but usually reduces the total training volume during this period of about 30% compared to regular training weeks, especially for elite athletes [20]. Although multiple studies have demonstrated the effectiveness of HIIT-SM to improve endurance performance [9–13] others have been less conclusive [16–19].

With a wide range of options for performing HIIT-SM, the optimal training volume to increase performance is a matter of debate. It is also unclear whether positive effects occur when additional training sessions are performed (i.e., additional low-intensity training (LIT)), as there is an anecdotal concern among practitioners that the overall lower training volume during HIIT-SM could lead to performance losses. Given this lower training volume and a training distribution towards high-intensity training during a HIIT-SM compared to standard training weeks, a novel approach with additional LIT added to the single HIIT sessions (not as separate training sessions) could shed light on the subject of the optimal training volume. The combination of LIT and HIIT applied in regular training weeks (microcycle/mesocycle) is widely accepted to improve endurance performance [4, 21–23].

Therefore, for the first time, we will conduct a randomized controlled trial (RCT) to compare the effects of two different types of HIIT-SM compared to a regular training regime on endurance performance in endurance trained athletes. One group will conduct a HIIT-SM with 10 HIIT sessions in 7 days (HSM), while the second intervention group completes the same HIIT-SM with an additional 30-min LIT after each HIIT session (HSM + LIT).

Besides the main outcomes relating to endurance performance, several secondary outcomes are addressed in this study. It is noticeable that the assessment of endurance performance in studies with a comparable design and training protocol vary between 3 and 9 days after completing a HIIT-SM [9–11, 13]. Consequently, it is unclear, when endurance performance generally peaks after a HIIT-SM and if there are individual response patterns that might be linked to kinetics of assessed variables during the training intervention. Repeated assessment of endurance performance at different time points after a HIIT-SM will address this issue and help practitioners optimally schedule HIIT-SM into athletes' competition schedules.

Another objective of our study is to identify monitoring assessments for load, recovery and stress. HIIT-SM require athletes to exert a great deal of effort due to repetitive HIIT sessions in a short period of time leading to muscular [24] and mental fatigue [25] as well as a transient inflammatory response as an acute response to HIIT [26]. Therefore, monitoring the athlete's external (e.g., power output (PO), running speed, distance covered) and internal load (e.g., biochemical markers, heart rate (HR), and questionnaires [27, 28]; but also the balance between stress and recovery [29–31] during these training periods is important to understand exercise-related fatigue to reduce the risk of injury or symptoms of overtraining [32]. Therefore, in this study we use a combination of subjective (e.g., questionnaires) and objective variables (e.g., biomarkers, neuromuscular performance) that provide us with a comprehensive picture of the athlete's training and corresponding psychophysiological response [33, 34].

Taken together, the aims of the present study are to (i) reveal the effects of two different types of HIIT-SM on endurance performance outcomes and (ii) identify suitable monitoring assessments for load, stress, and recovery in trained endurance athletes. In addition to introducing the study protocol, we will also provide the results of the first participant to illustrate the feasibility of our program and its extensive monitoring approach.

## Methods

### Experimental design

This study is registered (identifier: NCT05067426) and participant recruitment is currently ongoing. No results have yet been published on this study. The manuscript is in accordance with the SPIRIT guidelines (http://www.spirit-statement.org/) for reporting study protocols.

**Fig. 1:**
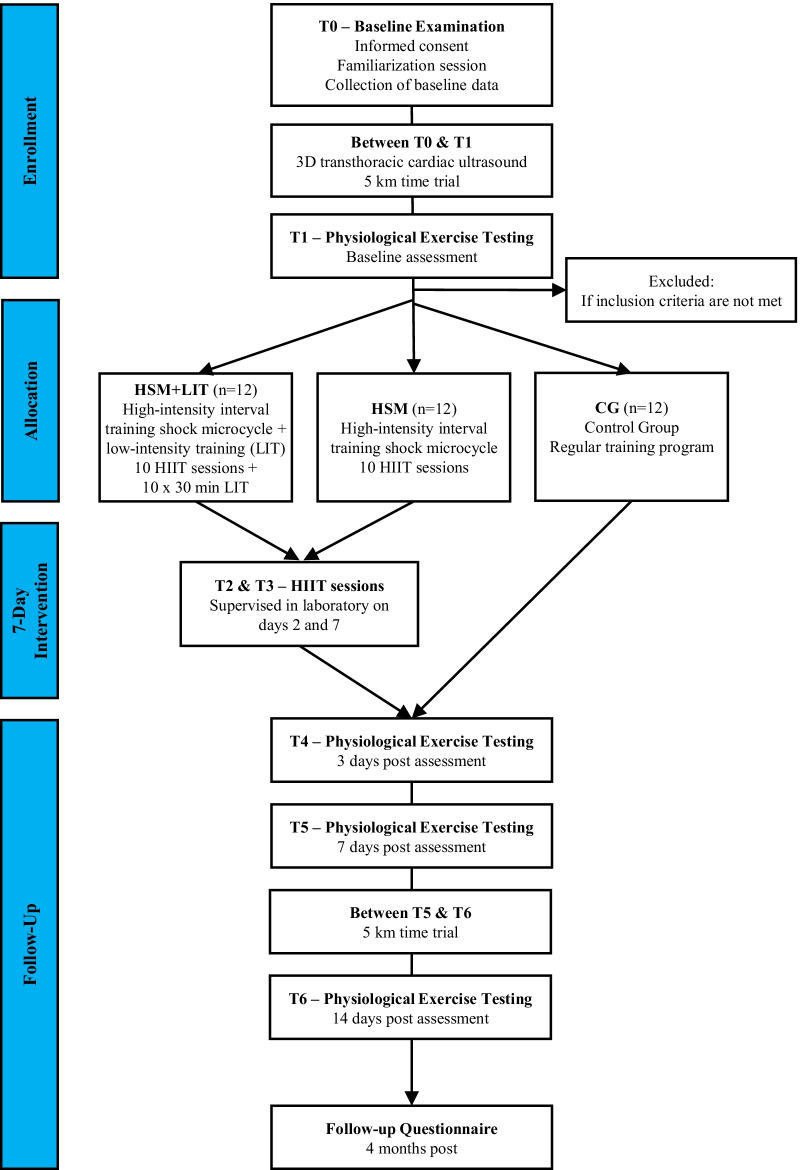
Study flowchart. The study is divided into a baseline period of 8–9 days (from T0 to T1), an intervention period of 7 days, and a post-intervention period of 14 days. Time points T0-T6 are face-to-face appointments for participants of all groups, with the HIIT sessions at T2 and T3 completed by the intervention groups, only

Protocol modifications (e.g., inclusion criteria, outcomes) are not intended. If these are nevertheless carried out, the responsible ethical board is informed with subsequent publication at clinicaltrials.gov. Study results will be published in peer-reviewed journals including presentations at symposia. The findings will be reported objectively regardless of the outcomes. All procedures have been approved by the local ethical board (University of Salzburg, GZ 2/2021) and are in conformity with the standards of the Declaration of Helsinki. The study may, if necessary, become the subject of an audit of the regional ethical board. The study is a single-center prospective RCT with three groups. Figure 1 outlines the study design.

In total, 36 participants will be randomly allocated at time point T1 by an unbiased researcher (NH) using a computer-generated allocation sequence, into either HSM, HSM + LIT or CG (balanced, one block with a ratio 1:1:1). Blinding is not applicable for participants after the allocation.

The total study duration is four weeks, consisting of a baseline phase (8–9 days), an intervention period (7 days), and a post-intervention phase (14 days). During the baseline phase, all participants complete baseline measurements (blood variables, resting electrocardiogram (ECG), questionnaires, neuromuscular performance, and executive functions) and become familiar with the equipment. The participants will continue with their regular training and report for entry physiological exercise testing at time point T1. Besides physiological exercise testing, the results of this test are used to determine the individual intensity ranges for the participants' HIIT sessions. Subsequently, participants of the HSM and HSM + LIT groups will perform 10 HIIT sessions during a 7-day intervention period. Four sessions are monitored in a laboratory setting, with two sessions at the second day of the HIIT-SM (T2) and the last two training sessions on the last day (T3). Following the intervention, a post-testing period of 14 days (with time points T4 after 3 days, T5 after 7 days, and T6 after 14 days) will evaluate the efficacy of the intervention. The CG continues their regular training and will report to time points T1, T4, T5 and T6.

During the study period, participants complete various assessments such as physiological exercise testing, 5 km time trial performance, questionnaires, blood sampling, neuromuscular, and executive function testing on a regular basis as described below in detail. During and 3 days post the intervention participants are not allowed to consume anti-inflammatory medications and recovery aids (e.g., massage, hot cold showers, pneumatic compression devices) to aid recovery. The consumption of food and food supplements will not be recorded. However, participants are encouraged to maintain their usual diet and not make dietary changes (e.g., consuming a new dietary supplement) throughout the study period. Tests are performed under controlled conditions supervised by experienced exercise scientists at the Red Bull Athlete Performance Center Thalgau, Austria and the University of Salzburg, Rif, Austria. Table 1 provides an overview of all included measures with the respective time points.

**Table 1:**
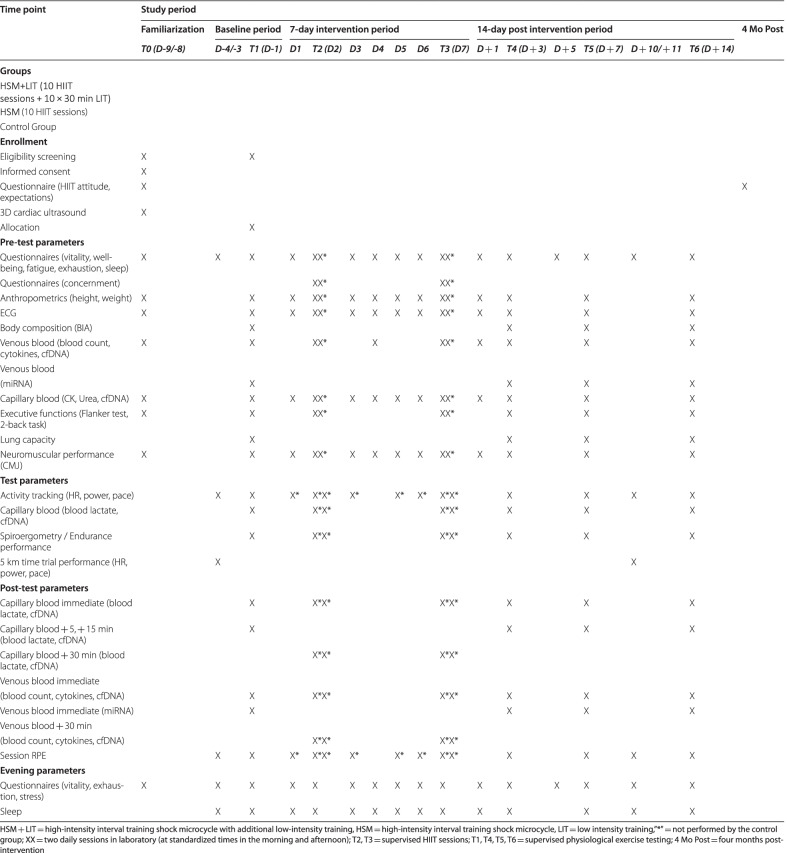
Overview of the included measures with the respective time points

### Participants

Thirty-six trained endurance athletes, either runners or athletes with a significant proportion of running as part of their regular training, aged 18–45 years with a maximal oxygen uptake (VO_2max_) of ≥ 50 ml/kg/min (female), or ≥ 55 ml/kg/min (male) [35, 36] or a 5 km time trial performance of ≤ 20:00 min (female), or ≤ 18:30 min (male) will be recruited via announcements on social media, through local sport clubs and universities (for detailed inclusion and exclusion criteria, please refer to Additional file 1 or clinicaltrials.gov). In advance, the participants will receive written information regarding the study. All participants are informed orally by the primary investigators (TS, JB) about the aims and risks (possible adverse events) of the study and must sign a written consent form prior to participation. The participants are allowed to leave the study at any time at their own discretion.

### Intervention

**Fig. 2:**
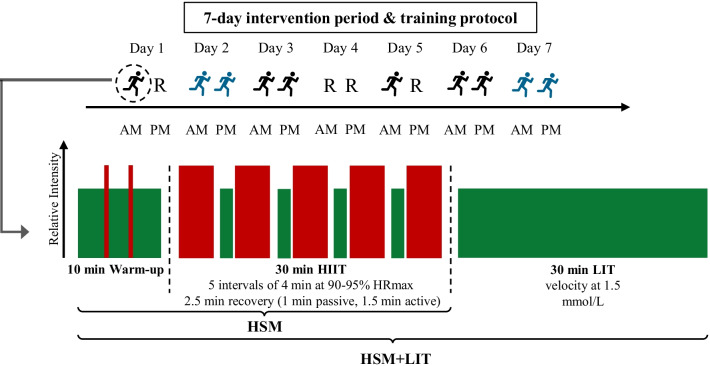
Outline of the 7-day intervention period and an exemplary training session. Outline of the 7-day intervention period (above) and an exemplary training session (below). Red bars represent intense runs and intervals at 90–95% HR_max_. Green bars represent warm-up, active recovery and 30 min LIT at a velocity at 1.5 mmol/L blood lactate. HIIT = high-intensity interval training, LIT = low-intensity training, HSM = high-intensity interval training shock microcycle, HSM + LIT = high-intensity interval training shock microcycle with additional low-intensity training, R = Rest, AM = morning, PM = afternoon, black runner = self-directed HIIT session, blue runner = supervised HIIT session

During the 7-day intervention period, participants of the HSM and HSM + LIT groups will complete a total of 10 HIIT sessions. All HIIT sessions will be performed with running and consist of 5 interval bouts of 4 min at an intensity eliciting at 90–95% of their individual maximum heart rate (HR_max_), interspersed by a 2.5-min recovery period (1 min passive rest for blood sampling and 1.5 min active recovery running at an intensity below 1.5 mmol/L blood lactate (La) determined at time point T1). Every session starts with a standardized 10-min low-intensity (velocity at 1.5 mmol/L La) warm-up program, which includes two intense runs of 30 s each (target speed of HIIT intervals at a velocity accordingly to 90–95% HRmax). This results in a total duration of 40 min per session. HIIT sessions are held in the morning (6 to 10 AM) and afternoon/evening (3 to 7 PM), with at least a 5-h break on days with two HIIT sessions. Figure 2 outlines the intervention period for both intervention groups.

In contrast to the HSM group, the HSM + LIT group will complete 30 min of LIT after each HIIT session (resulting in 300 min of additional LIT volume, i.e., 75% greater total training volume) at a velocity of 1.5 mmol/L La concentration as determined at T1. The HSM group is instructed not to perform an individual cool-down. All HIIT sessions are performed either self-monitored or under supervision on a treadmill (T2 and T3). During T2 and T3, spiroergometric data, and blood will be collected before, during HIIT and after HIIT/LIT. The intensity is controlled by continuous HR monitoring and adjusted if necessary. During the intervention participants of both intervention groups are instructed to complete no additional training sessions besides the prescribed HIIT.

### Outcomes

Primary objective: Evaluation of changes from baseline in endurance performance-related variables with respect to group, i.e.:

1. to determine the change of VO_2max_
2. to determine the change of peak performance in the VO_2max_ ramp test (P_peak_)
3. to determine the change of 5 km time trial performance
4. to determine the change of lactate threshold (LT)
5. to determine changes of fractional utilization of VO_2max_ at LT
6. to determine the change of running economy.

Secondary objective: Evaluation of changes from baseline in further variables with respect to group, i.e.:

1. to determine changes of questionnaire scores (stress, fatigue, sleep, vitality)
2. to determine changes of blood markers in acute (pre- vs. post-exercise) and chronic exercise (accumulated concentrations over time) settings (cell-free deoxyribonucleic acid (cfDNA), creatine kinase (CK), urea, La, cytokines, micro ribonucleic acid (miRNA))
3. to determine correlations of blood markers (cfDNA, CK, urea, La, cytokines, miRNA) with questionnaire scores and training variables (e.g., duration, distance, volume, power output, session rating of perceived exertion (Session RPE))
4. to determine the change of neuromuscular performance
5. to determine correlations of neuromuscular performance with training variables (e.g., duration, distance, volume, PO, session RPE)
6. to determine the change of body composition
7. to determine the change of pulmonary function variables
8. to determine the change of PO during physiological exercise testing and time trial
9. to determine the change of sleep quality
10. to determine the validity of different sleeping monitoring devices
11. to determine the change of resting ECG variables
12. to determine correlations of resting ECG variables with questionnaires
13. to determine the change of executive functions
14. to determine cardiac ultrasound variables of an athlete cohort
15. to determine adherence to the exercise programs (determined as training sessions performed: training sessions prescribed)
16. explorative analyses (such as data analysis about training intensity distribution, pattern recognition, analysis of individual training responses, further correlation analysis, machine learning approaches).

### Outcome measures

#### Physiological variables

**Fig. 3:**
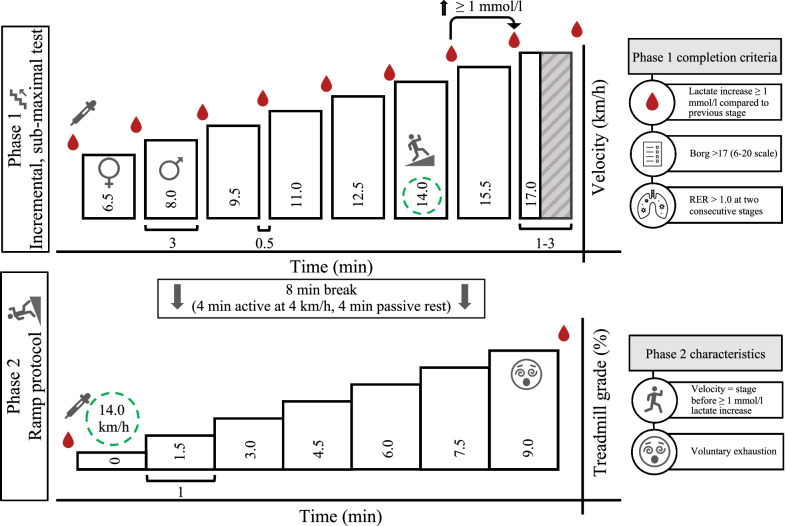
Illustration of the incremental, sub-maximal test (above) and ramp test (below) including completion criteria of phase 1 and phase 2 characteristics. Bars represent the duration, running velocity, and treadmill grade of each stage. Blood drop = capillary blood collection, ♀ = starting velocity for female participants, ♂ = starting velocity for male participants, green circle = determined velocity for phase 2 determined in phase 1, RER = respiratory exchange rate, min = minutes. During the incremental test, stages are three minutes each with an increment of 1.5 km/h per step. The ramp test stages last 1 min with a 1.5% increase in slope after each stage

*Endurance performance.* The efficacy of the intervention is measured by physiological exercise testing on a treadmill (Saturn, HP Cosmos, Traunstein, Germany) to determine VO_2max_ (Quark CPET, Comsed, Rome, Italy), P_peak_, and LT, as well as running economy and fractional utilization of VO_2max_ at LT. Participants will be instructed to refrain from strenuous exercise and alcohol for 24 h. Caffeine, food and drinks containing sugar should be avoided 3 h prior to all tests. Physiological exercise testing take place at time points T1, T4, T5 and T6 (Fig. 1) in the morning at a standardized time between 6 to 10 AM. The testing protocol used is a 2-phase test consisting of an incremental, sub-maximal exercise test (phase 1) followed by a ramp test (phase 2) interspersed with an 8 min break (4-min active walking at 4 km/h followed by 4 min passive rest; Fig. 3).

In brief, participants start the incremental test at 6.5 (female) or 8.0 km/h (male) at a treadmill incline of 0%. The increment is 1.5 km/h after every 3 min. The test is completed when either i) La has increased by ≥ 1 mmol/L compared to the previous stage [37], or ii) Borg value is > 17 (on the 6–20 scale [38]), or iii) the respiratory exchange ratio is > 1.0 in two consecutive stages. Criteria ii) and iii) were introduced to prevent participants who do not achieve a La increase of ≥ 1 mmol/L from becoming prematurely exhausted before the upcoming ramp protocol. The last stage is terminated when the result of the La level of the previous stage is displayed and being ≥ 1 mmol/L compared with the penultimate stage (generally 1 to 1.5 min). The speed of the ramp test is based on the results of the incremental test (equal to the speed of the stage before the La increase of ≥ 1 mmol/L) and remains constant with a steady increase in slope (1.5% per minute), starting at 0% until voluntary exhaustion without premature termination by the investigators. Based on the results of T1, the incremental test duration is matched at T4, T5, and T6. Consequently, the speed for the ramp test remains constant for all tests (T1, T4-T6) to achieve a high degree of comparability between tests.

**Fig. 4:**
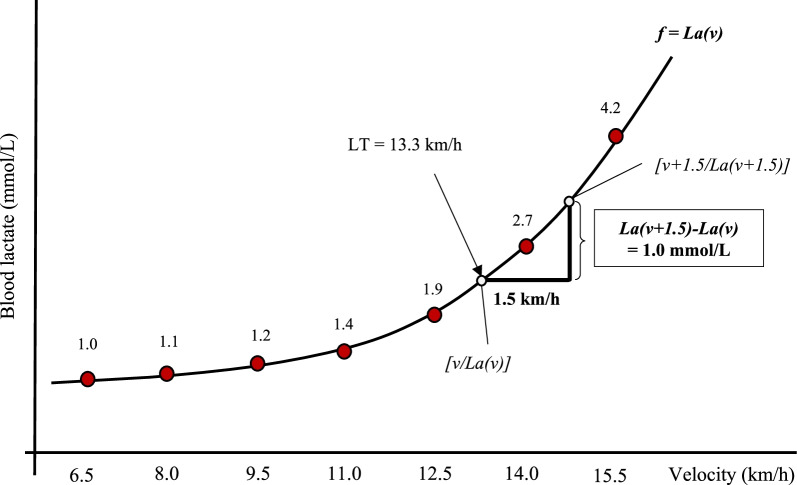
Lactate threshold determination. An example of how LT (lactate threshold) is determined based on the results of the incremental test. v, treadmill running velocity in km/h; f = best fit regression function of blood lactate over treadmill velocity; La(v), blood lactate values at the treadmill velocity v in mmol/L

During the breaks in the incremental test, pauses of approximately 30 s are used to collect capillary blood from the earlobe to determine La and blood glucose (Biosen S-line Clinic, EKF diagnostic GmbH, Magdeburg, Germany) sampling. Blood will also be collected at rest, between the incremental and the ramp test, and after the test. A modified version of the LT concept of Thoden [37] was introduced. Based on the criticism of an overly rough estimation of the LT (i.e., in 1.5 km/h steps), the following procedure will be performed: (1) the La—running velocity data is interpolated; best fit regression function: *f* = *La(v)* (with *La(v)* being the La value at a given running velocity v) and (2) based on the function *La(v)*, LT is defined as the velocity v where the delta value of *La (v* + *1.5)* and *La(v)* reaches first time 1 mmol/L La (e.g., *La(v* + *1.5) − La (v)* = *1*) (Fig. 4).

Spiroergometric data will be continuously analyzed using a breath-by-breath gas collection system. All respiratory data will be averaged every 5 s, and the highest 30 s rolling average during the ramp test will be defined as the VO_2max_. P_peak_ is calculated by linear interpolation using the formula: P_peak_ = %f + ((t/60) 1.5%), where %f is the treadmill grade of the last completed stage, t the duration of the last workload (s) and 1.5% the grade difference between the last two workloads.

*5 km time trial performance.* All participants will perform two self-directed 5 km time trials. The first time trial will be conducted before the intervention on day 6 or 7 during the baseline period, and the second one will be completed after the intervention on day 10 or 11 on the same outdoor course. Time, duration, distance, PO, and HR will be recorded.

*Lung capacity and body composition.* Prior to the physiological exercise testing (T1, T4, T5, T6), participants complete pulmonary function tests via spirometry (Cosmed, Rome, Italy) in a standing position (e.g., forced vital capacity, forced expiratory volume, peak expiratory flow). In addition, a bioelectrical impedance analysis (BIA; Seca, Hamburg, Germany) in the supine position with a hand-to-foot arrangement (4 electrodes) for body composition measures (e.g., body fat percentage, fat free mass, fat mass, skeletal muscle mass, total body water) will be conducted.

*Cardiac ultrasound.* All participants will undergo three-dimensional transthoracic cardiac ultrasound (3DTTE; EPIQ CVX, X5-1, Philips Healthcare, Andover, MA, USA) by a certified cardiologist with automated analysis of cardiac chamber volumes and functions (HeartModel, Philips Healthcare) prior to the first exercise test to screen for relevant cardiac pathologies and complete heart characterization. 3DTTE with automated cardiac volume analysis has shown to be accurate and reproducible [39], especially in individuals with good image quality, as regularly seen in an athletic population. Furthermore, automated cardiac function analysis facilitates assessment of exercise-induced cardiac adaptation in well-trained endurance athletes [40].

### Load, fatigue, and recovery monitoring

#### External load

*Activity tracking/training data.* To track activity type, time, and distance of all training sessions, participants will receive a Global Navigation Satellite System (GNSS) watch (Garmin Forerunner 935, Kansas City, MO, USA), a HR chest strap (HRM 3-SS, Kansas City, MO, USA), and a Stryd footpod (Stryd Wind V3, Stryd, Boulder, CO, USA). The Stryd sensor is used according to the manufacturer’s instructions and clipped to the subjects’ shoe laces to measure the athlete’s running PO during all runs, mean power output (MPO) during 5 km time trial and peak power output (PPO) during ramp test. A recent study found that the Stryd device is the most repeatable technology, among five analyzed systems, for running PO estimation [41]. Data are collected in the Garmin Connect application, which will be reviewed daily during the intervention by researchers to ensure program adherence. The training data of all groups is also recorded in a training log to record distance, and duration.

#### Internal load, fatigue, and recovery aspects

*Heart rate parameters and resting ECG*. During all training sessions a HR chest strap will record HR data to determine acute internal training load. A 10-min ECG (Norav medical, Mainz-Kastel, Germany) at rest in supine position will be written each day participants come to the laboratory. HR variability (HRV) will be evaluated via ECG, during sleep and a 3 min standardized short measurement in the morning via Abios (Algorithmic Biodata System GmbH, Salzburg, Austria).

*Sleep*. Sleep duration and quality will be recorded via Abios and the GNSS watch. Additionally, the watch tracks the rapid eye movement, light-, and deep-phases of habitual sleep.

*Neuromuscular performance.* Counter movement jump (CMJ) performance is suggested to be a measure of performance readiness and fatigue [24, 28, 42]. All participants will perform four maximal CMJ on a force platform (AMTI, Watertown, MA, USA) at T0–T6 and each day during the intervention after a standardized 5 min warm-up on the treadmill at a fixed speed and two warm-up trials. Average (best 3) CMJ height and further variables will be recorded in order to sensitively display neuromuscular performance respectively fatigue [43, 44].

*Blood parameters (venous)*. Differential blood count, venous cfDNA, ferritin, transferrin, circulating miRNA and various cytokine concentrations will be determined. Around 8 ml of blood will be obtained from the antecubital vein at T0-T6 at rest. At time points T1-T6, venous blood will also be drawn post-exercise. Inflammatory cytokines, such as Interleukin-6, interferon-γ and tumor necrosis factor-α, and other immunological markers (e.g., Interleukin-10) are determined using multiplex systems (Luminex® MAGPIX® System, Luminex Corporation, and flow cytometry, CytoFLEX S, Beckman Coulter). Both ferritin and transferrin concentrations will be determined using human enzyme-linked immunosorbent assay kits (Promocell and Invitrogen). Venous cfDNA will be quantified by analyzing unpurified plasma via quantitative real-time PCR (qPCR) as described elsewhere [45]. Circulating miRNAs will be isolated out of plasma samples (Macherey-Nagel and Qiagen) and certain miRNAs which have previously been shown to change in response to endurance exercise will be quantified via qPCR as previously described [46]. All blood samples will be centrifuged, and plasma and serum will be stored at > − 20 °C. Differential blood count will be determined using fresh whole blood by a Celltac MEK 6400 system (Nihon Kohden, Tokyo, Japan).

*Blood parameters (capillary)*. Capillary blood samples from the fingertip will be drawn at all study visits to determine cfDNA, CK, and urea concentrations at rest. At time points T1-T6, capillary blood will also be collected during and after physiological exercise testing and HIIT. cfDNA will be quantified using the above-mentioned protocol. Urea and CK will be determined via Reflotron Sprint (Roche, Basel, Switzerland). Lactate measurement is performed as described above.

*Questionnaires.* Validated questionnaires are completed prior to exercise (prior), directly post-exercise and at a standardized time of the evening (post) to cover various aspects of load, fatigue, vitality, stress, recovery and sleep duration and quality. Questionnaires are filled out by participants during baseline (three time points), the intervention (daily) and the post-intervention period (6 time points). At time point T0, participants will be asked about their overall attitude towards HIIT and HIIT-SM, as well as the expected outcome after conducting a HIIT-SM (adapted to Fuchs [47]). At all time points, vitality (prior and post, 4 items, Buchner et al., 2021, unpublished), and rating of fatigue (prior and post, 1 item, [48]) are recorded using a scale ranging from 0 to 10. Sleep (prior, 4 items) is assessed by rating sleep time, wake time, number of awakenings, and subjectively perceived sleep quality on a scale of 1–7 [49]. Moreover, muscular fatigue, muscular pain, and general well-being is assessed on a Visual-Analogue-Scale (VAS, i.e., a 100 mm line, endpoints are labeled accordingly with no pain, not fatigued, not strained on the left and extremely painful, maximal fatigued, extremely strained on the right). Scores will be determined from the distance in mm from the left border of the scale to the point marked [50]. During all days of the intervention, participants of both intervention groups will also rate their concern about the upcoming HIIT session from 0 to 10. Total training load will be determined 20 min after each training session by multiplying the category ratio RPE (from 1 to 10; [51]) scale, with the total duration of the training session [52].

Four months after study completion, a follow-up questionnaire with similar questions to T0 will be completed asking whether HIIT and HIIT-SM have become part of the participants’ regular exercise program.

*Executive functions.* At time points T0-T6, prior to exercise, participants will complete two tests which are measuring inhibition and working memory. Inhibition will be recorded through a modified Eriksen flanker task [53] consisting of 108 images showing five white arrows on a black screen. Working memory, is assessed through a 2-back task showing dots on a dice, numbers and geometrical figures. The applied tests are described elsewhere in detail [54].

#### Data management

JB, TS will be in charge of the data management. Identification is pseudonymous on all written and electronic files. All data entry and sensitive information regarding the participants will be password secured accessible only to the assigned researchers (JB, TS, TLS, NH). The data collection forms are available from the corresponding author on request 6 months after study completion.

#### Statistical analysis plan

Data will be collected using Excel (Microsoft Corp, Redmond, WA), and statistical analysis will be performed with SPSS (IBM, Chicago, IL, USA), R (RStudio, Inc., Boston, USA), GraphPad Prism (GraphPad Software, Inc, USA) and JMP (SAS Institute Inc, Cary, NC, USA) using standard statistical procedures. Data will be presented as mean ± SD or median (with inter quartile ranges) and are tested for normal distribution. In cases of non-normal distribution, data are log-transformed before statistical analysis to assess normality and variance homogeneity. A repeated-measure analysis of variance will be used to determine the differences between all groups and time points. Statistical significance will be set at alpha < 0.05. The magnitude of the changes between time points as well as the magnitude of differences in changes between the groups will be assessed using effect sizes (ES). Threshold values for ES are 0.2 (small), 0.6 (moderate), 1.2 (large), and 2.0 (very large) [55]. In addition, 90% confidence intervals for the between-groups differences in changes are estimated, and magnitude-based inferences are made with reference to a smallest worthwhile change, which is calculated as 0.2 multiplied by the between-subject variation at pretest [56]. The correlation between variables will be assessed using parametric (Pearson) and non-parametric (Spearman Rank) tests, depending on the distribution of the variables. In the case of multiple comparisons, p-values are adjusted using appropriate procedures such as the Bonferroni-Holm correction.

Adherence to the intervention will be defined as training performed: training prescribed in percentage. Data derived from participants discontinuing the intervention protocol or completing less than 90% (i.e., less than 45/50 intervals and/or less than 270/300 min LIT in HSM + LIT) of the prescribed training will be defined as dropouts. Participants will continue to be monitored at the time of withdrawal from the study if they agree. Depending on the parameter, dropouts are removed from the statistical analysis or enter the analysis using an intention-to-treat approach, for instance via data imputation using a mixed model approach.

A priori power analysis (repeated measures, within-between interaction) was done with G*Power (Version 3.1.9.7, Düsseldorf, Germany). Assuming a power of 0.8 and a medium effect (Cohen’s f = 0.25) on VO_2max_ (in line with [57, 58]), 27 participants are required. Thus, with a possible dropout rate of 20–25%, we will recruit 36 participants, or 12 participants per group.

We intend to complement the statistical analysis with a multidimensional and solely data-driven machine learning approach to characterize the athletes’ responses to HIIT-SM, using multivariate unsupervised machine learning models to address the data and variables according to their importance to the model, followed by assessing the predictive power of the variables identified concerning the athletes’ outcome to HIIT-SM.

## Case study

The following section presents the results of the first participant (male, assigned to HSM). At baseline, the participant reported to perform up to three endurance training sessions (60 min each) at moderate to high intensity and one strength training session (30 min) at high intensity per week. Adherence was 100%, i.e., the participant performed all 10 prescribed HIIT sessions and all corresponding intervals at the pre-determined intensity of 90–95% HR_max_ (171–181 bpm). Tables 2 and 3 provide an overview of the anthropometric data and the results of the performance variables.

**Table 2:** Anthropometric, spirometric and blood data of VP01 determined at T1 before the intervention

Age (years)	Weight (kg)	Height (cm)	BMI (kg/m^2^)	Body fat (%)	FVC (l)	FEV1 (l)	Hgb (g/dl)	Hct (%)
43	81.7	188.0	23.2	16.0	5.3	4.1	15.0	44.9

BMI = body mass index, FVC = forced vital capacity, FEV1 = forced expiratory volume in one second, Hgb = hemoglobin, Hct = hematocrit

**Table 3:** Presentation of changes in endurance performance variables

Endurance variable	Pre	Post		
	T1	T4	T5	T6
Incremental test				
LT (km/h)	11.9	12.1 (+ 1.7%)	12.4 (+ 4.2%)	12.4 (+ 4.2%)
HR at LT (bpm)	154	156 (+ 1.3%)	156 (+ 1.3%)	154 (+ 0.0%)
VO_2_ at LT (ml/min/kg)	39.1	39.8 (+ 1.8%)	39.8 (+ 1.8%)	40.7 (+ 4.1%)
VO_2_ at LT (%VO_2max_)	70.0	70.8 (+ 1.1%)	68.6 (− 2.0%)	70.4 (+ 0.6%)
Ramp test				
VO_2max_ (ml/min/kg)	55.8	56.3 (+ 0.9%)	57.5 (+ 3.0%)	57.8 (+ 3.6%)
HR_max_ (bpm)	191	188 (− 1.6%)	189 (− 1.0%)	190 (− 0.5%)
La_peak_ (mmol/L)	10.1	11.1 (+ 9.9%)	11.2 (+ 10.9%)	12.6 (+ 24.8%)
PPO (W)	470	489 (+ 4.0%)	496 (+ 5.5%)	503 (+ 7.0%)
TTE (min:s)	7:25	8:05 (+ 9%)	8:20 (+ 12.4%)	8:35 (+ 15.7%)
Time trial				
5 km (min:s)	21:29		20:50 (− 3.0%)	
HR mean (bpm)	181		180 (− 0.6%)	
MPO (W)	349		368 (+ 5.4%)	

VO_2max_ = maximal oxygen uptake, HR_max_ = maximal heart rate, La_peak_ = peak blood lactate, PPO = peak power output determined via Stryd, TTE = Time to exhaustion, LT = lactate threshold according to the modified version based on Thoden, 1991, HR at LT = heart rate at lactate threshold, VO_2_ at LT(ml/min/kg) = oxygen uptake at lactate threshold, VO_2_ at LT (%VO_2max_) = fractional utilization of VO_2max_ at lactate threshold, 5 km time trial = self-directed in outdoor environment, HR mean = mean heart rate, MPO = mean power output determined via Stryd

As shown in Table 3, the participant achieved marked improvements in endurance performance, i.e., VO_2max_, PPO, VO_2_ at LT, TTE, and time trial performance. For some variables, such as VO_2max_, the improvement does not seem to have occurred yet three days after the intervention rather at a later time point, which needs to be investigated with a larger sample and also with the second intervention group.

**Fig. 5:**
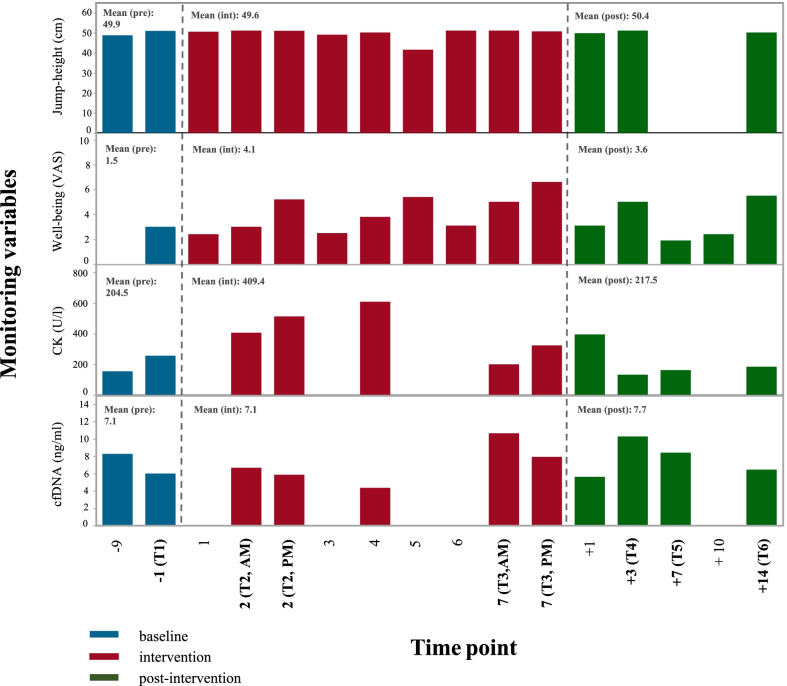
Overview of selected monitoring variables. An overview of selected variables (y-axis; neuromuscular performance, well-being, CK, cfDNA) at time points (x-axis) at rest. The VAS (well-being) scores should be understood such that a higher score indicates a decrease in well-being. Biomarker monitoring in the present case was measured at selected time points during the intervention phase and will be extended to a daily assessment in the upcoming study

Figure 5 outlines selected variables of our load monitoring approach using biomarkers, questionnaires, and neuromuscular performance.

CMJ height remained relatively constant, whereas mean CK and well-being scores increased markedly during the intervention with peak scores/concentrations on day 4 and day 7, respectively. Mean cfDNA concentrations were similar during the intervention compared to baseline but showed an increase towards the end of the intervention compared to the beginning.

## Discussion

To the best of our knowledge, this will be the first study to investigate the effects of two HIIT-SM with different total training volumes on VO_2max_ and other endurance relevant variables such as time trial performance, and LT compared to a control condition.

Previous studies have shown that HIIT-SM significantly increases aerobic performance [9–13, 59], with the majority of studies using long intervals to improve the aerobic capacity. Therefore, in this study, participants are required to perform a total of 10 HIIT sessions within one week using a highly standardized 5 × 4-min program with 2.5 min of recovery (and 30 min of LIT for HSM + LIT). Interventions with a slightly longer duration but a comparable number of sessions using a similar aerobic HIIT training program demonstrated both feasibility and improvements in endurance performance [9, 11]. Thus, performance improvements of 3–6% regarding VO_2max_ as well as additional improvements in LT, time trial, and running economy are highly likely for both HIIT-SM groups [60].

The widely adopted idea of polarized training [4, 21, 22], i.e., a combination of LIT and HIIT, leads us to assume that further adaptations may occur in HSM + LIT. LIT serves as a potent stimulus to enhance fat oxidation and glucose utilization, which are essential for aerobic energy provision during prolonged endurance training [61–64]. In contrast, HIIT may lead to cardiopulmonary improvements, such as increased stroke and blood volume [2, 65]. In addition, metabolic alterations [66] such as increased mitochondrial biogenesis and oxidative capacity are possible [67–69]. Both training methods lead to an increased capacity for aerobic adenosine triphosphate generation in endurance athletes when practiced regularly with sufficient recovery between sessions [22]. It remains to be seen whether the combination of HIIT and LIT in such a condensed HIIT-SM shows superior effects on endurance performance compared to a HIIT-SM that relies on HIIT as the main training stimulus (HSM group). It cannot be ruled out that the additional training volume may even have negative effects such as performance decline, or injury.

The 14-day follow-up period with repeated physiological exercise testing at days 3, 7, and 14 after the intervention allows us to assess when performance is restored or maximized, as there is uncertainty on this issue after two versions of HIIT-SM. For instance, Hatle et al. [12] showed that a “high frequency group” (24 HIIT sessions in three weeks) achieved no significant improvement four days after the intervention on VO_2max_. The highest value in this group was achieved 12 days post-intervention. In contrast, the “moderate frequency group” (24 HIIT sessions in eight weeks) achieved their P_peak_ directly after the intervention. Compared to the pretest, both groups were able to maintain their improved VO_2max_ up to four weeks after the intervention [12]. These results indicate that adaptations following a HIIT-SM may occur in different trajectories depending on the training dose. The timing of VO_2max_ assessment seems thus crucial for the identification of meaningful changes.

HIIT-SM are very challenging for athletes due to the high intensity of training over a longer period of time. This can lead to a decrease in well-being, higher perceived exertion, muscular fatigue, mental fatigue, and slower recovery during and after a HIIT-SM [25, 70]. Athletes have difficulties reaching prescribed HR zones towards the end of the intervention compared to the beginning as cumulative fatigue and disturbance in the autonomic nervous system suppresses HR_max_ [9, 33, 71]. The second important outcome will therefore be to assess the suitability of different load monitoring tools to accurately track aspects of external load, internal load, fatigue, sleep, and recovery. Monitoring these aspects during exhaustive training periods is critical to keep the risk of injury, illness, and overtraining low [32].

According to general consensus both external and internal load should be assessed using subjective and objective measures [28, 32, 34]. Our approach using tracking devices, questionnaires, biomarkers, neuromuscular performance, and executive functions will help understand how periods of strenuous training translate into changes in internal load, stress and recovery, well-being, cognitive performance, and sleep to obtain a holistic picture of the load of the athlete. In addition to established variables such as HR, CK, or questionnaires, innovative markers such as various cytokines or cfDNA concentrations will be investigated for the first time. This approach aims to establish further useful and reliable markers for monitoring athletes during high-intensity training periods. Biomarkers, in particular cfDNA, have demonstrated their potential as variables for training load in a variety of acute and chronic conditions [72–76].

### Feasibility

Our case study demonstrated the feasibility and safety of our study protocol as well as high participant adherence leading to a performance increase compared to baseline (Table 3). Interestingly, some performance variables (VO_2max_, TTE) showed a progressive improvement after the intervention (from T4 to T6); an observation that is interesting to investigate further. Various internal load parameters (Fig. 5) provide a comprehensive picture of the athlete’s fatigue and recovery over the course of the study. However, the results of this case study should be interpreted with caution. A conclusion on the suitability of novel biomarkers, such as cfDNA, for monitoring purposes can only be made with a higher sample size. In the upcoming study, aspects of external and internal load, fatigue, and recovery will be measured on a daily basis during the intervention to monitor participants even more closely.

### Limitations

In previous HIIT-SM studies, the training program was predominantly performed under supervision, in contrast to our study, which provides self-directed and supervised training sessions. We are aware that performing HIIT might cause difficulties in meeting predefined intensity ranges and volumes, potentially limiting the adherence. However, all participants need to be familiar with tracking their training sessions using wearable devices (e.g., GNSS watch, HR monitor, Stryd) and training logs. Data from these training sessions are reviewed on a daily basis by researchers to confirm that participants train at predefined intensities and volumes. Another aspect is that no further training specifications are provided to participants during the baseline phase and the 14-day post-intervention period. While this represents regular training habits, it also leads to uncertainty about the effects of individual training volumes and their impact on subsequent performance assessments. However, due to three physiological exercise tests and the time trial in the post-intervention period and the associated restriction not to train on the day before, the factor of individual training should be negligible. In our study, participants voluntarily log their menstrual cycles (onset and end of menstruation), but exercise and performance assessments are not adjusted for the phases of the menstrual cycle. Therefore, the data does not allow to answer whether this affects aerobic performance and recovery. In addition, differences in dietary behavior between participants, e.g., carbohydrate consumption [77, 78] or intake of dietary supplements such as caffeine [79], could affect performance and recovery. Given that we advised participants to refrain from changes in dietary behavior, this is likely to have a rather small effect on the results. Finally, it should be noted that, apart from a follow-up questionnaire after four months, the study does not include a long-term performance assessment to determine sustained benefits of the intervention.

## Conclusion

This study will provide highly relevant insights into physiological and psychological load during and adaptations following two versions of HIIT-SM and highlight potential differences in adaptations between different training volumes. Here, we are testing for the first time a novel approach that has previously only been applied in meso-, or macro-cycles where HIIT and LIT are performed generally in separate training sessions. In addition, new innovative tools for monitoring load, stress and recovery will potentially emerge from our study, as our approach covers various aspects of external and internal load.

## Supplementary Information

**Additional file 1.** Eligibility criteria.

## Data Availability

The dataset used and analyzed during the current study is available from the corresponding author on reasonable request.

## Abbreviations

HIIT: High-intensity interval training
SM: Shock microcycle
HIIT-SM: High-intensity interval training shock microcycle
LIT: Low-intensity training
HSM: High-intensity interval training shock microcycle (intervention group 1)
HSM + LIT: High-intensity interval training shock microcycle with additional low-intensity training (intervention group 2)
CG: Control group
RCT: Randomized controlled trial
HR: Heart rate
PO: Power output
ECG: Electrocardiogram
VO_2max_: Maximal oxygen uptake
HR_max_: Maximal heart rate
La: Blood lactate
P_peak_: Peak performance
LT: Lactate threshold
CK: Creatine kinase
cfDNA: Cell-free deoxyribonucleic acid
miRNA: Micro ribonucleic acid
RPE: Rating of perceived exertion
La(v): Blood lactate at treadmill velocity
BIA: Bioelectrical impedance analysis
3DTTE: Three-dimensional transthoracic cardiac ultrasound
GNSS: Global Navigation Satellite System
PPO: Peak power output
MPO: Mean power output
HRV: Heart rate variability
CMJ: Counter movement jump
qPCR: Quantitative real-time polymerase chain reaction
VAS: Visual analogue scale
VO_2_: Oxygen uptake
