# Supplementary material for: The Salzburg 10/7 HIIT shock cycle study: the effects of a 7-day high-intensity interval training shock microcycle with or without additional low-intensity training on endurance performance, well-being, stress and recovery in endurance trained athletes—study protocol of a randomized controlled trial
Source: BMC Sports Sci Med Rehabil. 2022 May 7;14:84. doi: 10.1186/s13102-022-00456-8 (PMC9077880; doi:10.1186/s13102-022-00456-8)
Supplement: Supplementary file 1 — Additional file 1. Eligibility criteria. [file 13102_2022_456_MOESM1_ESM.docx]

**Eligibility Criteria**

| Ages Eligible for Study: | 18 Years to 45 Years |
| --- | --- |
| Sexes Eligible for Study: | All |
| Gender Based: | No |
| Accepts Healthy Volunteers: | Yes |

**Criteria**

Inclusion Criteria:

- Female or male
- aged 18-45 years
- Proof of physical fitness (e.g., sports medical examination required) for measurements with higher intensities (e.g., endurance tests, competition simulation)
- Competition experience at the national or international level in an endurance sport
- VO2max ≥50ml/kg/min for females; ≥ 55 ml/kg/min for males or 5 km time trial performance of ≤ 20:00 min (female), or ≤ 18:30 min (male)

Exclusion Criteria:

- Systemic disease or other known pathology in the organs: heart, lungs, kidney, stomach, spleen, liver, gall bladder, and intestines.
- Evidence of pulmonary disease: forced expiratory volume in one second/forced expiratory volume < 70% with/without symptoms (cough, sputum) or other evidence of pulmonologic disease.
- Diabetes II.
- Neurological or psychological disease of any kind.
- Currently undergoing medical or psycho-therapeutic treatment.
- Health condition that does not allow regular participation in the training forms (e.g. acute illnesses such as fever or other flu-like infections within the last 7 days before the start of the study), orthopedic diseases, injuries to the muscular, bone, joint or tendon apparatus within the last three months.
- Alcohol or drug abuse.
- Already high training volume with high intensity training (more than 2 weekly training sessions of high-intensity training)
